# Cardiac magnetic resonance assessment of right ventricular remodeling after anthracycline therapy

**DOI:** 10.1038/s41598-021-96630-y

**Published:** 2021-08-24

**Authors:** Thiago Ferreira de Souza, Thiago Quinaglia Silva, Lígia Antunes-Correa, Zsofia D. Drobni, Felipe Osório Costa, Sergio San Juan Dertkigil, Wilson Nadruz, Fabrício Brenelli, Andrei C. Sposito, José Roberto Matos-Souza, Otávio Rizzi Coelho, Tomas G. Neilan, Michael Jerosch-Herold, Otávio Rizzi Coelho-Filho

**Affiliations:** 1 Division of Cardiology, Department of Medicine, Faculdade de Ciências Médicas - Universidade Estadual de Campinas (UNICAMP), Rua Tessália Viera de Camargo, 126, Campinas, SP CEP 13083-887 Brazil; 2 Massachusetts General Hospital, Harvard Medical School, Boston, MA USA; 3 Noninvasive Cardiovascular Imaging Program and Department of Radiology, Brigham and Women’s Hospital, Boston, MA USA

**Keywords:** Breast cancer, Cardiac hypertrophy, Predictive markers

## Abstract

There are limited data on the effects of anthracyclines on right ventricular (RV) structure, function, and tissue characteristics. The goal of this study was to investigate the effects of anthracyclines on the RV using cardiac magnetic resonance (CMR). This was a post-hoc analysis of a prospective study of 27 breast cancer (BC) patients (51.8 ± 8.9 years) using CMR prior, and up to 3-times after anthracyclines (240 mg/m^2^) to measure RV volumes and mass, RV extracellular volume (ECV) and cardiomyocyte mass (CM). Before anthracyclines, LVEF (69.4 ± 3.6%) and RVEF (55.6 ± 9%) were normal. The median follow-up after anthracyclines was 399 days (IQR 310–517). The RVEF reached its nadir (46.3 ± 6.8%) after 9-months (*P* < 0.001). RV mass-index and RV CM decreased to 13 ± 2.8 g/m^2^ and 8.13 ± 2 g/m^2^, respectively, at 16-months after anthracyclines. The RV ECV expanded from 0.26 ± 0.07 by 0.14 (53%) to 0.40 ± 0.1 (*P* < 0.001). The RV ECV expansion correlated with a decrease in RV mass-index (r = −0.46; *P* < 0.001) and the increase in CK-MB. An RV ESV index at baseline above its median predicted an increased risk of LV dysfunction post-anthracyclines. In BC patients treated with anthracyclines, RV atrophy, systolic dysfunction, and a parallel increase of diffuse interstitial fibrosis indicate a cardiotoxic response on a similar scale as previously seen in the systemic left ventricle.

## Introduction

Anthracyclines are a standard primary therapy for several malignancies. However, the use of anthracyclines is associated with cardiac injury, adverse cardiac remodeling, and cardiac dysfunction leading to clinical heart failure (HF) and death^1^. Multiple prior studies^2–5^ have provided an in-depth characterization of the changes of left ventricular (LV) and aortic vascular function in patients treated with anthracyclines. Among broad groups of patients with HF, the presence and the extent of right ventricular (RV) dysfunction is a key determinant of outcomes^6–8^, but there are limited data on the effect of anthracyclines on the RV^9,10^. In patients treated with anthracyclines, studies using CMR imaging have demonstrated a decline in RV systolic function in adult survivors of childhood cancer^11,12^ and in breast cancer patients^13–15^. However, one of the key strengths of CMR is the ability to apply tissue characterization techniques to improve our understanding of the pathophysiological changes in response to injury. There are a paucity of studies applying such tissue characterization techniques to better understand the effects of anthracyclines on the RV. This is in contrast to several published studies on LV tissue remodeling after anthracyclines^2–5^; additionally, several studies^16,17^ have suggested that the RV may be more vulnerable to injury from anthracyclines, and early RV dysfunction may predict anthracycline-induced cardiotoxicity. We were specifically interested in understanding the effects of anthracyclines on the RV fibrosis, as measured by calculation of the extracellular volume (ECV), and on cardiomyocyte mass. Such data would improve the current understanding of pre-clinical RV abnormalities induced by anthracycline therapy in BC patients, and are hypothesis generating for future prospective studies of the long-term effects of anthracycline induced cardiotoxicity.

## Methods

### Study design

This is a post-hoc analysis of a previously published clinical study applying CMR to characterize the cardiotoxicity with anthracyclines (ClinicalTrials.gov: DOX-0675014600011)^2^ in 27 consecutively recruited female patients with BC enrolled between 2012 and 2015. We previously described^2^ the effects of anthracyclines in this study on the LV. Patients were eligible for enrollment if they had a BC diagnosis and were scheduled to receive adjuvant anthracycline-based therapy (doxorubicin in 4-cycles at 60 mg/m^2^, total dose of 240 mg/m^2^). Exclusion criteria were any contraindications to CMR, chronic kidney disease (GFR < 40 ml/min^2^), previous myocardial infarction, clinical diagnosis of HF, moderate or severe valvular disease or any other significant cardiac disease. Detailed clinical and medical history, standard anthropometric data, and laboratory evaluation were performed alongside CMR exams. Our local Institutional Review Board approved the study (CAAE: 0675.0.146.000-11, Comitê de Ética em Pesquisa (CEP) da Faculdade de Ciências Médicas da UNICAMP; Rua: Tessália Vieira de Camargo, 126; Distrito de Barão Geraldo, Campinas—SP, Brazil, CEP: 13083-887; https://www.prp.unicamp.br/pt-br/contato-2; Phone/Fax: + 55 19 3521.8936). All participants provided written informed consent prior to study enrollment and patients completed the study protocol without suffering adverse events. All methods were carried out in accordance with relevant guidelines and regulations.

### Follow-up

Participants underwent clinical assessment and CMR imaging before and up to three times consecutively after anthracycline treatment (median follow-up time points were: 140, 231, and 427 days from initiation of anthracyclines for visits 1, 2, and 3, respectively, as previously reported^2^. Time ranges for study visits and CMR exams varied mainly due to patient scheduling and retention issues. Thus, for the longitudinal analysis, we categorized the follow-up times into quartile ranges. In the three patients with HER-2 + breast cancer, trastuzumab was delayed for non-study related reasons and was not administrated until after the last CMR exam.

### Biochemical analysis

Blood samples were obtained at baseline and during each anthracycline cycle and before each CMR examination. Glucose, glycated hemoglobin, triglycerides, high and low-density lipoprotein cholesterol (Beckman Coulter, AU5800 Beckman Coulter Analyzer, United States) were obtained at baseline after 12 h of fasting. CK, CK-MB (Beckman Coulter, AU5800 Beckman Coulter Analyzer, United States) and high-sensitive troponin T (cTnT) (Roche, Cobas e601 immunoassay analyzers, Roche Diagnostics, Germany) were obtained at baseline, before each anthracycline cycle, and before each CMR visit.

### Cardiac magnetic resonance

Patients were imaged on a 3 T CMR scanner (Achieva, Philips Medical Systems, The Netherlands) with a **6**-element phased-array surface-coil. The CMR protocol comprised electrocardiographically gated cine imaging with steady-state free precession (repetition time TR = 3.4 ms; echo time TE = 1.2 ms; in-plane spatial resolution = 1.5 mm) for LV and RV volumes/function, and imaging of late gadolinium enhancement (LGE) for scar assessment as previously described^18^. A breath-hold Look-Locker spoiled gradient-echo cine technique^19^ was used for T1 measurements in a single mid-ventricular slice with a temporal resolution of 80 ms for pre-contrast T1 and 55 ms post-contrast (TR/TE/flip-angle = 5/2.2 ms/10°; slice thickness = 8 mm; 192 × 128 matrix; FOV =  ~ 340 × 300–340 mm; NEX = 1; SENSE = 2 parallel imaging acceleration, in-plane resolution of 1.7 × 1.7 mm)^20^. The Look-Locker sequence used an adiabatic inversion pulse, which by its very nature is relatively insensitive to the B1 amplitude of the radio-frequency pulses and results in excellent inversion profiles. At least 5 T1 datasets were acquired in each patient.

### Assessment of biventricular volumes, function, fibrosis by CMR

The CMR images were analyzed with the MASS CMR software (Mass Research, Version 2021, Leiden University Medical Center, Leiden, the Netherlands, www.lkeb.nl) to measure biventricular mass, volumes and function^21^. The RV and LV walls were contoured on the Look-Locker images and divided into 4 and 6 segments, respectively. Ventricular volumes and mass were indexed by body-surface area. For the RV ECV, only the free wall from the superior to the inferior RV-to-LV insertions points was considered. T1s were determined for each myocardial segment and the LV blood pool by nonlinear least squares fitting to an analytic expression for the inversion recovery and correction for the effects of radiofrequency pulses applied using the method originally developed by Deichman and Haase^22^. The myocardial partition coefficient for gadolinium contrast (λ_Gd_) was determined by myocardial sector by linear least-squares fitting of pre and post-contrast R1’s in tissue against R1’s in blood. ECV was estimated as λ_Gd_ multiplied by (*1-Hct*), where *Hct* represents the patient’s blood hematocrit at the time of the CMR examination^23^. We also estimated the LV and RV cardiomyocyte mass (CM) from the product of (1-ECV) and total LV and RV mass, respectively.

### Statistical analyses

Statistical analysis was performed using R (version 4.0.3, R Foundation; http://www.R-project.org/). Data are presented as means ± standard deviation, or median with inter-quartile range (IQR), if not normally distributed. Linear mixed effects (LME) regression models (R-package *lme4*) were used to analyze longitudinal changes of LV and RV volumes indices, EF and ECV in each patient. Measurement variables which did not appear to have a linear dependence on follow-up time (as continuous variable) were analyzed with generalized additive models with mixed effects (GAM), using a cubic-regression spline to represent time^24^, a random intercept per patient, and an autoregressive model (AR) for errors to account for the correlation of successive measurements within each patient (R-package *mgcv*). Variables were log-transformed as needed when a quantile–quantile plot indicated significant deviation from a normal distribution. The occurrence of LV systolic dysfunction after anthracycline therapy was stratified by the median RVEF or RV ESV index at baseline. Bivariate correlation of independent measurements was assessed with Pearson’s method, or alternatively with Spearman’s method if indicated. Repeated measures correlation was computed to determine the overall within-individual relationship among paired measures assessed at multiple time points^25^. Intra and inter-observer variability of the RV ECV was performed at patient and segmental levels in a randomly selected subset of studies (n = 10), using inter-class correlation (ICC)^26^. The subset of studies was re-analyzed blinded to any clinical information by the main reader (TFS) and by another reader (ORCF), both fully trained in CMR and with prior experience in T1 mapping analysis.

## Results

### Baseline clinical and imaging characteristics of the study population

Baseline characteristics of our study population are shown in Table 1. None of the study participants had diabetes mellitus or history of angina or myocardial infarction. Before anthracyclines, all subjects had normal LVEF (69.4 ± 3.6%), LV mass index (51.4 ± 8 g/m^2^) and RV mass index (18.2 ± 4 g/m^2^)^27,28^. The mean RVEF at baseline was 55.6 ± 9%, consistent with values reported for healthy normal^29^ of similar age.

**Table 1:** General characteristics.

**Demographics and clinical data at baseline**	
Age	51.8 ± 8.9
Body mass index, kg/m^2^	26.9 ± 3.6
Body surface area, m^2^	1.8 ± 0.1
Heart rate	72 ± 11
Hypertension, %, (N)	22.2 (6)
Diabetes, %, (N)	0 (0)
Hyperlipidemia, % (N)	14.8 (4)
Tobacco current use, %, (N)	22.2 (6)
Former smoker, %, (N)	11.1 (3)
Framingham 10-year risk for a CVD event, % (range)	5 (1.4–21.1)
The Framingham age adjusted risk increment, % (range)	2.9 (0–17.7)
**Medication use**	
Angiotensin-converting enzyme inhibitor, %, (N)	14.8 (4)
Aspirin, %, (N)	3.7 (1)
*β*-Blocker, %, (N)	3.7 (1)
Statin, %, (N)	14.8 (4)
Metformin, %, (N)	0 (0)
Insulin, %, (N)	0 (0)
**Laboratory analyses**	
HDL-cholesterol, mg/dl	59 ± 14.9
LDL-cholesterol, mg/dl	144 ± 28
Triglycerides, mg/dl	120 ± 35
CRP levels, mg/l	0.3 ± 0.2
Glucose, mg/dl	89 ± 12.9
Glycated hemoglobin, %	5.5 ± 0.3
White blood cells, 10^3^/mm^3^	6.1 ± 0.9
Glomerular filtration rate, ml/min/1.73 m^2^	125 ± 35

Data are presented as mean ± SD, n (%) or median (range) when appropriated. *CVD*  cardiovascular disease.

### Changes in standard LV and RV morphology and function after anthracycline-based chemotherapy

**Figure 1:**
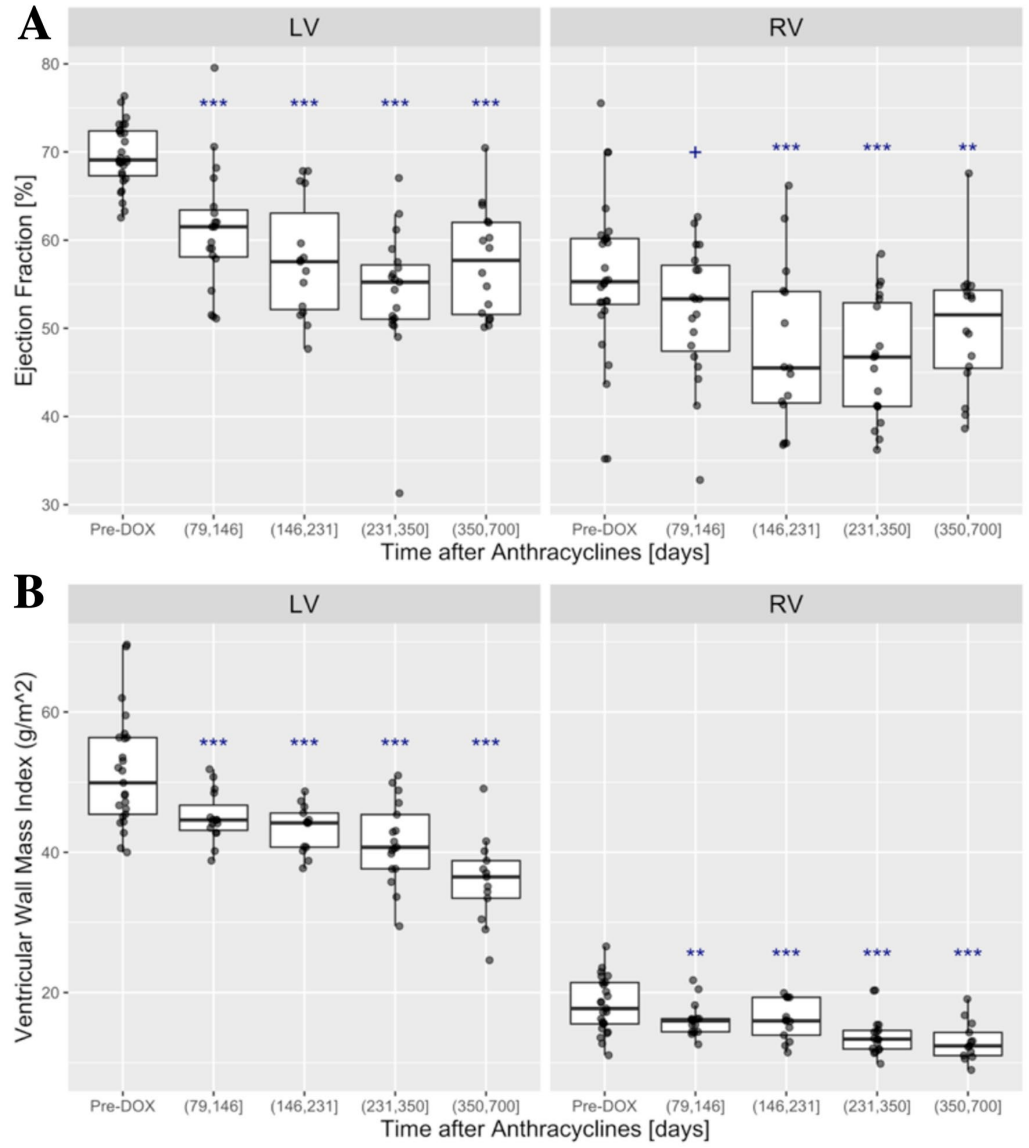
AB: Baseline and post-anthracycline ejection fraction (LV and RV) and ventricular mass (LV and RV). (**A**) LV EF (left) was significantly lower during all follow-up periods after anthracycline therapy (*P* < 0.001); RV EF (right) reached a minimum around (146, 231] days after anthracycline initiation (*P* = 0.002 vs. pre-DOX), followed by a slight recovery. (**B**) LV and RV ventricular mass index showed steady incremental decreases over the entire follow-up and in all follow-up periods both LV and RV mass index were significantly (*P* < 0.001) below their baseline averages (^+^*P* < 0.1; *P < 0.05; **P < 0.01; ***P < 0.001).

The median follow-up time after anthracyclines was 399 days (IQR 310–517) and covered a time span from 79 to 700 days. Measurements for RV and LV volumes, wall mass and systolic function are summarized in Fig. 1 and the supplemental online Table 1. The quartile ranges for follow-up time were: (79, 146], (146, 231], (231, 350], and (350, 700] days relative to the initiation of anthracycline therapy, corresponding to median follow-up times of 4, 6, 9, and 16 months, respectively. As described previously^2^, the LVEF and LV mass-index decreased from baseline, reaching 53.8 ± 8.4% at (231, 350] days (median 9 months) and 36 ± 6 g/m^2^ at (350, 700] days (median 16 months) after chemotherapy respectively (*P* < 0.001; Fig. 1A,B).

The RV end-diastolic volume index did not change after anthracyclines (supplemental online Table 1). In contrast, there was an approximately 25% increase in the RV end-systolic volume index from 20.4 ± 6 ml/m^2^ at baseline to 25.2 ± 10 ml/m^2^ at (146, 231] days after anthracycline therapy (*P* < 0.011). This increase in the RV end-systolic volume occurred with a parallel decline in the RVEF from 55.6 ± 9% to a minimum of 46.3 ± 6.8% at (231, 350] days after anthracyclines (*P* < 0.001 compared with baseline, Fig. 1A). The RV mass index decreased by 28% after anthracyclines, from 18.2 ± 4 g/m^2^ at baseline to 13 ± 2.8 g/m^2^ at a median of 16 months after chemotherapy (*P* < 0.001 compared with baseline, Fig. 1B), similar to a parallel 30% decrease of LV mass index. The RVEF correlated only weakly (repeated measures correlation coefficient r = 0.23, *P* = 0.05; CI [− 0.012, − 0.44] with the LVEF.

### Right ventricular extracellular remodeling after anthracycline therapy

**Figure 2:**
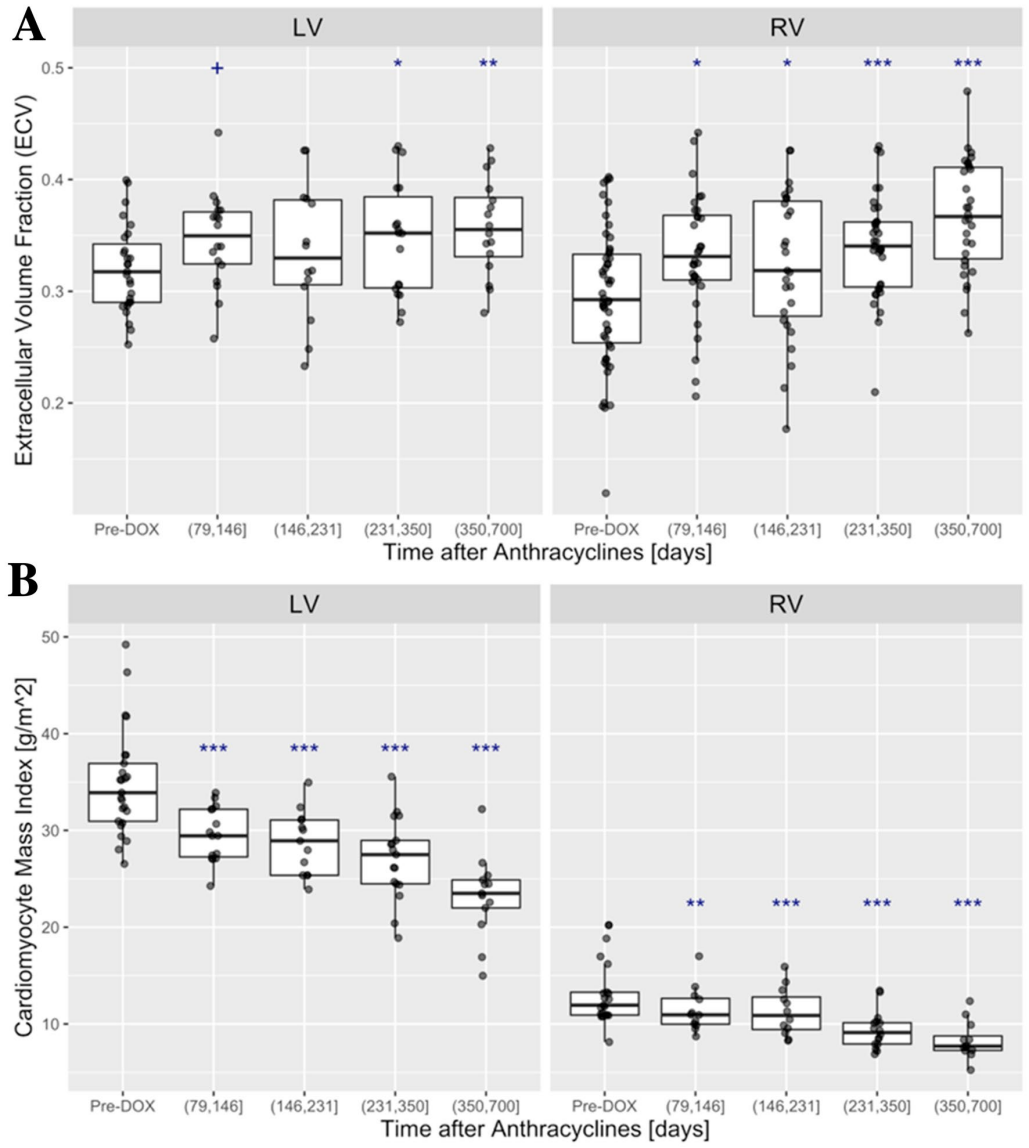
Baseline and post-anthracycline LV and RV of the ECV and cardiomyocyte mass index (CM): (**A**) LV ECV (left) increased from 0.32 ± 0.04 at baseline, by 0.037 (11%), to 0.36 ± 0.04 in the (350, 700] day period (*P* = 0.0035 for (350, 700] days vs. baseline). The RV ECV (right) also increased from the baseline value of 0.26 ± 0.07 by 0.14 (53%) to 0.40 ± 0.1 in the (350, 700] day period (*P* < 0.001 for (350, 700] days vs. baseline). (**B**) LV and RV cardiomyocyte mass decreased steadily after anthracyclines (^+^*P* < 0.1; *P < 0.05; **P < 0.01; ***P < 0.001).

Paralleling the longitudinal expansion of ECV in the LV (Fig. 2A), the RV ECV also increased from a baseline value of 0.26 ± 0.07 by 0.14 (53%) to 0.40 ± 0.1 in the (350, 700] day period (*P* < 0.001 for (350, 700] days vs. baseline, Fig. 2A). There was no significant correlation between the LV and RV ECV’s (rep. meas. r = 0.19; *P* = 0.14). Both the LV and RV cardiomyocyte mass (CM), derived from the product of (1-ECV) and wall mass indices, decreased after chemotherapy, reaching 23.2 ± 4.3 g/m^2^ for the LV and 8.3 ± 1.93 g/m^2^ for the RV, respectively, at (351–700] days after chemotherapy (Fig. 2B). The RV ECV correlated weakly with the RV mass-to-ED volume (repeated measures correlation coefficient r = 0.33; *P* = 0.012; CI [− 0.54, − 0.07]).

### Changes in serum biomarkers

**Figure 3:**
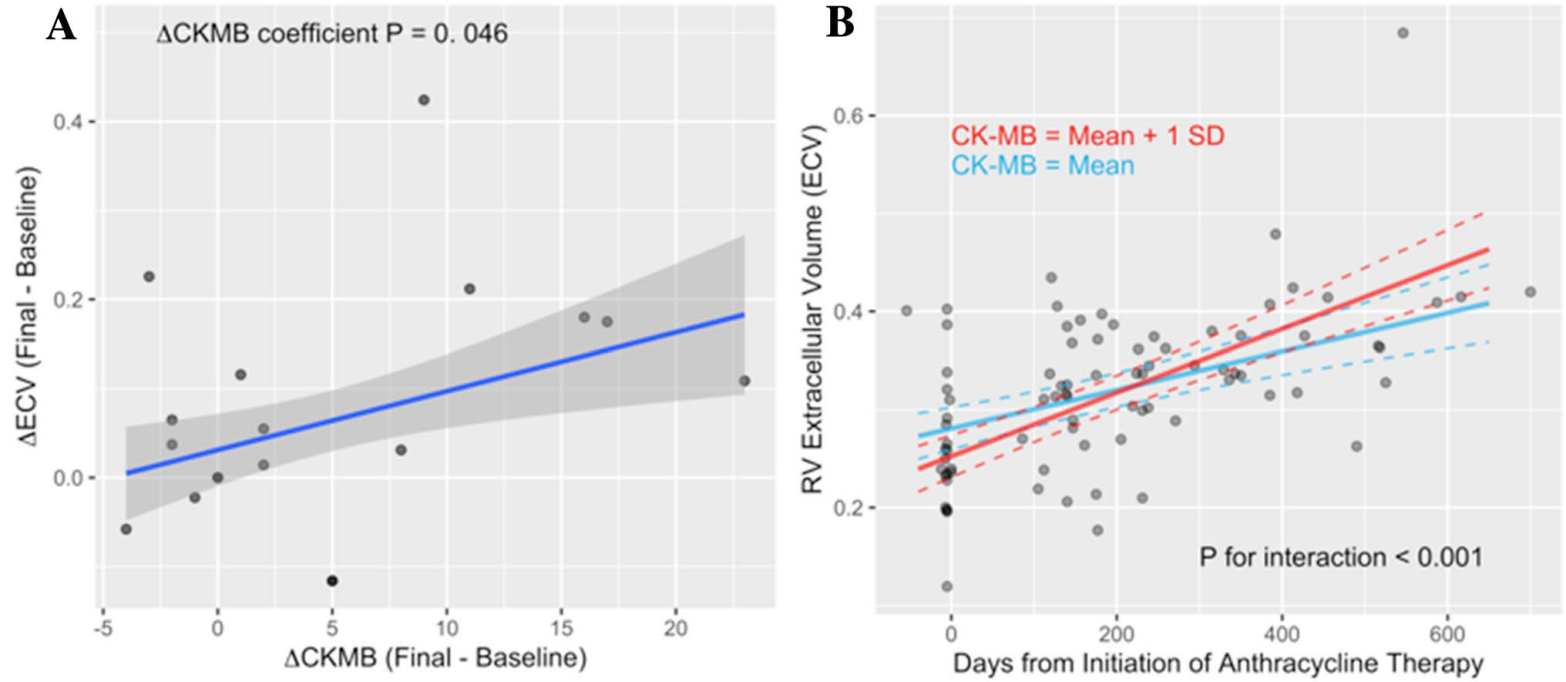
Changes of RV ECV and their association with CK-MB. (**A**): the change of ECV in the RV between the baseline MRI study and the final CMR (ΔECV) in each patient correlated with the change of CK-MB (ΔCK-MB) a serum biomarker of myocardial injury. (**B)** RV ECV increased approximately linearly with time from anthracycline therapy. The rate of RV ECV expansion over time after anthracycline therapy (*P* < 0.001 for follow-up-time coefficient estimate) increased in proportion to the serum level of CK-MB activity (*P* < 0.001 for interaction of follow-up time with log-transformed CK-MB level). The longitudinal change of RV ECV was modeled with a linear mixed effects model that included follow-up time (days), log-transformed CK-MB and an interaction term as predictors. The blue and red lines show the predictions from this model for two levels of CK-MB corresponding to the overall mean (blue) of CK-MB and a one standard deviation higher level (red), respectively.

Results for the serum biomarkers cTnT and CK-MB are displayed in the online supplement (supplemental online Table 2). As previously reported^2^, the cTnT and CK-MB fraction levels increased from 4.6 ± 1.4 pg/ml and 13.6 ± 6.1 IU/L (baseline) to 21.3 ± 14.4 pg/ml and 19.8 ± 11.7 IU/L at (79,146] days after doxorubicin administration, respectively. Thereafter, the cTnT and CK-MB declined to 5.2 ± 1.6 pg/ml at (350, 700] and 15.8 ± 8.5 IU/L at (231, 350] days after chemotherapy. While patients with peak cTnT > 10 pg/ml had a more prominent decrease of LV CM after anthracyclines, this observation was not replicated by RV CM. The repeated measures correlation of RVEF and CK-MB was − 0.36 (*P* = 0.005). The change of RV ECV between baseline and the final follow-up CMR study was associated with the simultaneously measured change of CK-MB serum level, as shown in Fig. 3A. Furthermore, the longitudinal change of RV ECV was modified by CK-MB levels: patients with higher CK-MB had a larger change of ECV (*P* < 0.001 for interaction of follow-up time and log-transformed CK-MB level), as illustrated in Fig. 3B. The change of hs-cTnT between baseline and the final follow-up CMR study was not associated with the change of RV ECV.

### Reproducibility analyses of the RV ECV

Reproducibility analyses of the RV ECV was performed in a randomly selected subset of exams (n = 10). The inter-class correlation (ICC) of RV ECV at segmental and patient levels were performed for inter-observed and intra observer variability analysis. The ICC coefficient for the inter-observer comparison at the segmental level was 0.795 (CI 0.678–0.873) and at the patient level was 0.784 (CI 0.373–0.94). The ICC for intra-observer comparison of the RV ECV at segmental level was 0.82 (CI 0.717–0.888) and at patient level was 0.927 (CI 0.473–0.953).

### Baseline RV function as predictor of left ventricular dysfunction post anthracycline

**Figure 4:**
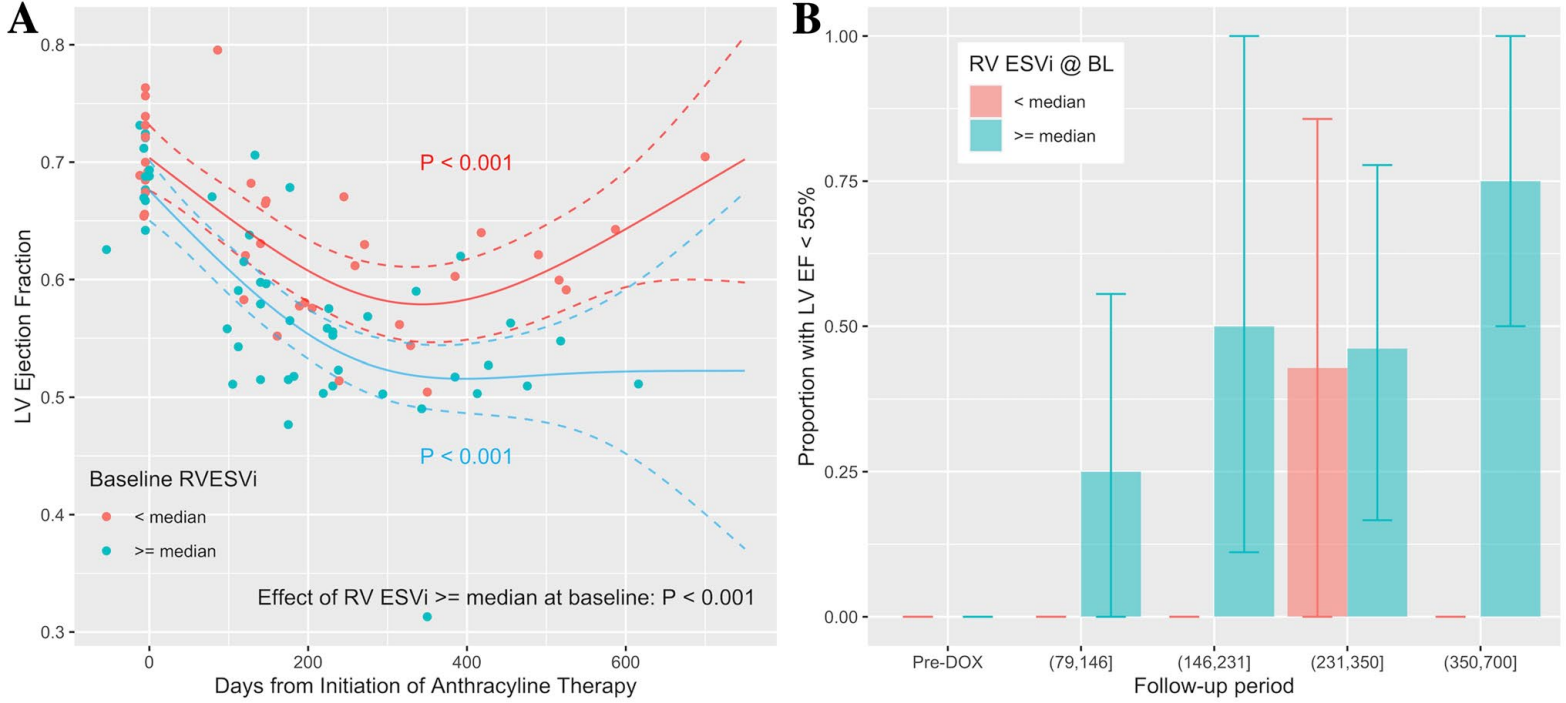
Predictors of LV systolic dysfunction post-anthracycline: (**A**) LVEF was within the normal range in all patients at baseline and declined significantly after anthracycline therapy (*P* < 0.001). After anthracyclines, LVEF was significantly lower in patients with higher than median RV ESVi at baseline compared to in patients with an RV ESV index < its median (55%) at baseline (BL). The solid lines represent cubic regression splines for the time from anthracycline therapy initiation, which were estimated with a generalized additive model (GAM) with mixed effects. The model also included RV ESVi at baseline as independent predictor. The effect of an above median RV ESVi at baseline remained significant (*P* < 0.001) when LV ESVi at baseline was added to the model. (**B)** The bar graph presents an analysis complementary to A for the proportion of patients with LVEF < 55% during follow-up periods. LV EF < 55% was significantly more frequent during follow-up if RV ESV index ≥ median at baseline—the final follow-up period from > 350 to 700 days, the proportion reached 75% percent. At baseline, all patients with RV ESVi < median had an LVEF within the normal range. The error bars denote the 95% confidence intervals and were generated by the bootstrap method with sampling by patient identifier.

After anthracycline therapy, 15 (58%) patients showed signs of LV systolic dysfunction (LVEF < 55%; mean decrease of LVEF in group developing LV dysfunction: 18.5 ± 5.3%; min. change 11.5%) on one of the follow-up CMR examinations, i.e. these patients met criteria for LV cardiotoxicity, namely a > 10% reduction in LVEF from baseline to < 55%, in the absence of heart failure symptoms. Patients with LV systolic dysfunction at follow-up had a lower RVEF at baseline (51.49 ± 7.72 vs. 60.82 ± 8.91%; *P* = 0.011) and a higher RV ESVi (23.66 ± 6.15 vs. 17.20 ± 4.07 ml/m^2^; *P* = 0.005). Patients with an RV ESVi above its median at baseline, had an *overall* lower LVEF at *multiple* time points after anthracyclines (*P* < 0.001), though at baseline LVEF was > 60% in all patients (Fig. 4A). In patients with RV ESVi ≥ median at baseline, LV systolic dysfunction was more likely after anthracyclines (*P* = 0.0028 for effect of RV ESVi ≥ median at baseline, Fig. 4B). Similarly, patients with an RVEF below its median at baseline, had an *overall* lower LVEF at *multiple* time points after anthracyclines (*P* < 0.001).

## Discussion

This study provides the first evidence of anthracycline-induced adverse myocardial remodeling of the RV manifested by RV atrophy, and expansion of the extracellular volume (ECV). The ECV is a well-validated imaging marker of myocardial fibrosis in the LV and increased following anthracycline therapy; however, has not been used previously to study cardiotoxicity in the RV. The simultaneous increase of ECV and a reduction in total RV wall mass both contributed to a reduction of RV CM, suggesting CM atrophy and/or diffuse CM necrosis. Furthermore, subclinical differences in RV systolic EF and end-systolic volumes at baseline predicted LV systolic dysfunction after anthracyclines. These findings are consistent with recent reports that the RV may be at least as vulnerable to injury from anthracyclines as the LV, and early RV dysfunction may predict anthracycline-induced cardiotoxicity^16,17^. In addition, the reproducibility analysis for the RV ECV, demonstrated that our measurements were reliable, consistent and reproducible with ICC coefficients > 0.75^26^.

A decline in RV systolic function is a robust prognostic marker for adverse outcomes in a variety of clinical scenarios^8,30–34^. Nevertheless, there are limited data on the effect of anthracyclines on the RV. In the Interagency Registry for Mechanically Assisted Circulatory Support (INTERMACS), 27% of patients presented with chemotherapy-induced cardiomyopathy and these patients had significant and severe reduction of RVEF^35^. Only a few CMR studies have characterized the occurrence of RV dysfunction after anthracyclines with or without antagonism of human epidermal growth factor receptor 2 (HER2)^13–15^. Groover et al. noted, in cohort of 56 BC patients treated with anthracycline and/or trastuzumab, a significant decrease in RVEF at 4 months, which persisted to 12 months^13^. Nakano and colleagues, studying exclusively HER positive BC patients, reported changes on the RV circumferential strain, which decreased 6-months after the initiation of trastuzumab^14^. More recently, Barthur et al. also investigated HER positive BC patients before, and serially after chemotherapy (6, 12, and 18 months), and showed adverse effects on RV structure and function that tended to recover after 18 months^15^. Recently Zhao et al.^36^, reported in a cohort with large B-cell lymphoma, treated with anthracycline-based chemotherapy, that the RVEF decreased significantly from 54.0 ± 2.8% to 49.8 ± 2.4% (*P* < 0.001), a change of a similar magnitude as in the present study.

Although criteria for RV cardiotoxicity are not well-defined or agreed upon, it has been proposed that significant reductions of RVEF below the lower limit of normal for the imaging method should be considered^36^. Since the lower normal limit of RVEF has been defined as 51% for women^28^, our findings highlight that even moderated exposure to anthracycline may cause a meaningful decline in RV function. Furthermore, an above-median RV ESV index and a below-median RVEF at baseline were both independently associated with a higher risk of LV cardiotoxicity after anthracyclines (> 10% decline of LV EF and LV EF < 55%). RV dysfunction may even represent an early warning sign of cardiotoxicity following anthracycline therapy. A previous study by Planek et al. of lymphoma patients^16^ with an average follow-up period of 6-months found that anthracycline therapy was associated with subclinical RV dysfunction, but not LV dysfunction, at a cumulative dose ≥ 200 mg/m^2^. In the present study LV systolic dysfunction was at a nadir around ~ 300 days allowing a more comprehensive evaluation of full impact of anthracyclines on ventricular function than in the study by Planek et al. A novel finding from this study is that the RV ESVi at baseline is associated with the risk of LV systolic dysfunction after anthracycline therapy, suggesting that RV ESVi baseline status could help in risk stratification for patients undergoing anthracycline therapy.

We observed a relatively weak correlation between the longitudinal changes of EF in the LV and RV. In prior work, RV remodeling and dysfunction was noted at 4–6 months after therapy implementation and lasted until 12-months reflecting or not LV changes in function^13–15^. Experimental models of mice treated with doxorubicin demonstrate that histopathologic modifications in ventricular tissues are analogous, but the degree by which each ventricle is affected may differ, particularly in relation to the level of oxidative stress present^37^. Our group^38^, and others^39^, have also demonstrated that anthracycline-based chemotherapy markedly reduces LV mass, and that this portends a worse prognosis. The mechanisms involved in the decrease in LV mass are thought to be related to cardiomyocyte death and atrophy^2^. It is reasonable to hypothesize that cardiomyocyte death and atrophy play a similar putative role in the RV loss of mass.

Several groups^2–4^ have demonstrated the usefulness of CMR T1 mapping in characterizing myocardial tissue remodeling of the LV in patients undergoing cancer therapy, but there are no analogous data for the RV. T1 mapping in the RV is technically more challenging. Nevertheless, studies in patients have demonstrated the value of CMR T1 mapping for detecting diffuse fibrosis in the RV. We note here that the RV ECV at baseline in our study (0.26 ± 0.07) is almost identical to the RV ECV reported for control groups in two independent studies of pulmonary hypertension (0.264 ± 0.042^40^, and median 0.271 with IQR = (0.251–0.279)^41^). There are limited data from experimental studies on longitudinal RV ECV changes following anthracycline therapy. In a rabbit model^42^, the ECV increased at both superior and inferior RV insertion regions from 0.28 ± 0.013 and 0.29 ± 0.02, to 0.39 ± 0.01 and 0.41 ± 0.01, respectively, 16 weeks after treatment with doxorubicin (1 mg/kg injections twice a week). The T1-based ECV estimates at the RV insertion regions correlated well with the collagen volume fraction (r = 0.824, *P* < 0.001)^42^.

## Limitations

We have investigated a relatively small uniform cohort of BC patients; consequently, current results may not be applicable to patients with a different malignancy clinical profile. As all recruited patients remained asymptomatic during the entire study, exploratory investigation of the associations of RVEF and RV ECV with symptoms after anthracycline therapy was not feasible. In addition, we highlight that we used a Look-Locker technique for T1 measurements rather than the modified Look-Locker imaging (MOLLI), as this sequence was not available at the time of the study. A generalization or comparison of our results obtained with the Look-Locker gradient echo T1-mapping method, to other more commonly used T1 mapping methods, may not apply. It should nevertheless be noted that MOLLI and Look-Locker T1 measurements showed good agreement^43^. Other biomarkers of interest, such as NT-proBNP, were not initially planned and were not available. There was a negative correlation between RVEF and CK-MB, and it appears plausible that a similar relationship could be expected between RV EF and hs-cTnT, but which was nevertheless not observed. We note here that CK levels, including of the CK-MB heterodimer, have been reported to be lower^44^ in breast cancer patients, and more so with more advanced cancer stage, which may result in a larger longer-term effect of anthracyclines on CK-MB levels compared to hs-cTnT. Finally, it is important to acknowledge that the current findings have to be interpreted in the context of a hypothesis generating post-hoc study which requires further, larger and dedicated investigations to confirm the clinical usefulness of RV tissues characterization by CMR T1 mapping following anthracyclines.

## Conclusion

Breast cancer patients undergoing anthracycline-therapy experienced significant adverse myocardial remodeling in both ventricles, which are reflected in meaningful reductions in function and mass. Similar to the LV, the RV extracellular volume increased and RV cardiomyocyte mass decreased after anthracycline therapy, expanding the current understanding of RV dysfunction induced in this setting.

## Supplementary Information

Supplementary Tables.

## Abbreviations

BC: Breast cancer
CK: Creatine kinase
CK-MB: Creatine kinase myocardial band fraction
CM: Cardiomyocyte mass
CMR: Cardiovascular magnetic resonance
cTnT: High-sensitive troponin T
ECV: Extracellular volume
FOV: Field of view
GAM: Generalized additive models with mixed effects
GFR: Glomerular filtration rate
Hct: Hematocrit
HER2: The antibody human epidermal growth factor receptor 2
HF: Heart failure
IQR: Inter-quartile range
LGE: Late gadolinium enhancement
LME: Linear mixed effects
LV: Left ventricle
MOLLI: Modified Look-Locker imaging
NEX: Number of excitations
RV: Right ventricle
TE: Echo time
TR: Repetition time
λ_Gd_: Partition coefficient for gadolinium contrast
