# Supplementary material for: Cardiac magnetic resonance assessment of right ventricular remodeling after anthracycline therapy
Source: Sci Rep. 2021 Aug 24;11:17132. doi: 10.1038/s41598-021-96630-y (PMC8385101; doi:10.1038/s41598-021-96630-y)
Supplement: Supplementary file 1 — Supplementary Tables. [file 41598_2021_96630_MOESM1_ESM.docx]

Online-only **Data Supplement:**

**Online Tables:**

**Online Table 1: Baseline and Follow-Up CMR Findings (**online-only **Data Supplement):**

| Median days after anthracycline | Pre-DOX | (79,146] | (146,231] | (231,350] | (350,700] |
| --- | --- | --- | --- | --- | --- |
| N | 27 | 16 | 19 | 14 | 16 |
| LVEF, % | 69.4±3.6 | 61.1±7.6^†^ | 56±5^†^ | 53.8±8.4 ^†^ | 57.5±6.1 ^†^ |
| LVEDV index, ml/m^2^ | 60.2±9.9 | 64.3±9.6 | 66.7±17.7 * | 56.9±18.5 | 59.2±12.6 |
| LVESV index, ml/m^2^ | 18.3±4.0 | 24.7±7.3 ^#^ | 29.0±7.3 ^&^ | 26.2±9.7 ^&^ | 25.3±8.2 ^&^ |
| LV mass index, g/m^2^ | 51.4±8.0 | 45.3±3.8 ^&^ | 43.2±4.9 ^&^ | 39.9±5.4 ^&^ | 36.0±6.1 ^&^ |
| LV ECV | 0.32±0.04 | 0.34±0.04 | 0.33±0.06 | 0.35±0.05 * | 0.36±0.04 ^#^ |
| LV cardiomyocyte mass, g/m^2^ | 34.9±5.5 | 29.6±2.8 ^&^ | 28.8 ± 3.7 ^&^ | 27.1±4.3 ^&^ | 23.2±4.3^&^ |
| RVEF, % | 55.6±9 | 52.1±7.5 | 47.7±9.3^#^ | 46.3±6.8^&^ | 50.2±7.3* |
| RVEDV index, ml/m^2^ | 47.2±9.4 | 51±12.4 | 47±12.8 | 45±14.3 | 48±11.6 |
| RVESV index, ml/m^2^ | 20.4±6 | 24.4±6.9 | 25.2±10* | 24±8.6^#^ | 22.9±6.3 |
| RV mass index, g/m^2^ | 18.2±4 | 15.8±2.6^#^ | 15.7±3^&^ | 14±2.9^&^ | 13±2.8^&^ |
| RV ECV | 0.26±0.07 | 0.37±0.21^#^ | 0.33±0.06* | 0.35±0.04^#^ | 0.40±0.1^#^ |
| RV cardiomyocyte mass, g/m^2^ | 13.8±3.33 | 11.48±2.29* | 11.28±2.45* | 9.37±1.94^#^ | 8.3±1.93^&^ |

Data are presented as mean ± SD. LV: left ventricle; RV: right ventricle; EF: ejection fraction; EDV: end diastolic volume; ESV: end systolic volume (average/SD).

*Significantly different from Pre-DOX level (p<0.05 from linear mixed effects model).

^#^ Significantly different from Pre-DOX level (p<0.01 from linear mixed effects model).

^&^ Significantly different from Pre-DOX level (p<0.001 from linear mixed effects model).

**Online Table 2: Baseline and Follow-Up Biochemical Analysis (**online-only **Data Supplement):**

| Median days after anthracycline | Pre-DOX | (79,146] | (146,231] | (231,350] | (350,700] |
| --- | --- | --- | --- | --- | --- |
| N | 27 | 16 | 19 | 14 | 16 |
| CK-MB fraction, U/L | 13.6 ± 6.1 | 19.8 ± 11.7 * | 19.1 ± 8.5 * | 15.8 ± 8.5 | 17.2 ± 7.8 |
| hs-CRP, mg/L | 0.3 ± 0.2 | 0.4 ± 0.5 | 0.3 ± 0.2 | 0.12 ± 0.7 * | 0.3 ± 0.4 |
| hs-Troponin T, ng/dL | 4.6 ± 1.4 | 21.3 ± 14.4 * | 10.7 ± 3.2 * | 6.6 ± 3.7 * | 5.2 ± 1.6 |
| Hemoglobin, g/dL | 13.1 ± 0.9 | 12.1 ± 1.0 * | 12.1 ± 1.0 * | 12.1 ± 0.8 * | 12.1 ± 1.0 * |
| Hematocrit, % | 39.8 ± 2.5 | 36.4 ± 2.8 * | 36.5 ± 2.8 * | 36.1 ± 2.6 * | 37.1 ± 2.8 * |

Data are presented as mean ± SD. CK: Creatine kinase; CRP: C Reactive Protein; hs: high-sensitivity; (average/SD).

*Significantly different from Pre-DOX level (p<0.01 from linear mixed effects model).
